# Glucose metabolism and endometrium decidualization

**DOI:** 10.3389/fendo.2025.1546335

**Published:** 2025-02-17

**Authors:** Yunfei Huang, Qinling Zhu, Yun Sun

**Affiliations:** ^1^ Department of Reproductive Medicine, Ren Ji Hospital, Shanghai Jiao Tong University School of Medicine, Shanghai, China; ^2^ Shanghai Key Laboratory for Assisted Reproduction and Reproductive Genetics, Shanghai, China; ^3^ Center for Reproductive Medicine, Ren Ji Hospital, Shanghai Jiao Tong University School of Medicine, Shanghai, China

**Keywords:** decidualization, glucose metabolism, GLUTs, glycolysis, Warburg effect

## Abstract

Prior to embryo implantation, the endometrial stromal cells (ESCs) during the menstrual cycle undergo a significant structural and functional transformation known as decidualization to support conception. During this process, glucose consumption and utilization by endometrial cells increase to meet energy demands. Abnormal glucose metabolism in the endometrium impairs decidualization, leading to pregnancy complications, including implantation failure and pregnancy loss. However, the mechanisms modulating glucose metabolism in endometrial stromal cells during decidualization are still unclear. In this review, we describe the functions and regulation of glucose transporters (GLUTs) involved in glucose uptake, as well as the modulation of key enzymes catalyzing glucose utilization. Moreover, we present recent findings on the role of glucose related metabolites in the decidualization of ESCs.

## Introduction

**Figure 1:**
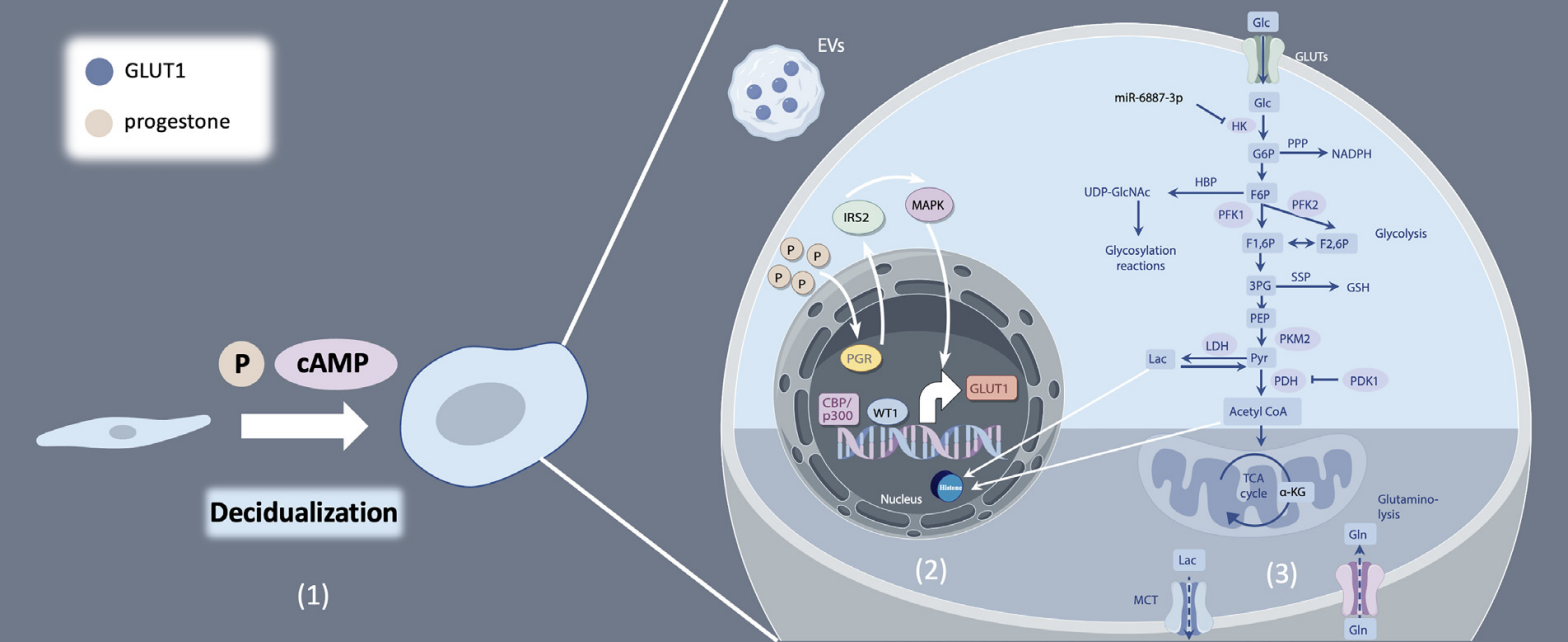
Decidualization and glucose metabolism of endometrial stromal cells. (1) Endometrial stromal cells (ESCs) differentiate into decidual stromal cells (DSCs) under the influence of progesterone, as well as the accumulation of intracellular cyclic adenosine monophosphate (cAMP). (2) Multiple regulation mechanisms of GLUT1. (3) Glucose catabolism and modulation from uptake to utilization.

Endometrial decidualization is a prerequisite for successful embryo implantation. Decidualization is the process by which endometrial stromal cells (ESCs) differentiate into decidual stromal cells (DSCs) under the influence of progesterone and the accumulation of intracellular cyclic adenosine monophosphate (cAMP) (**Figure 1**). This process enables the endometrium to acquire a receptive phenotype, promoting embryo implantation and pregnancy maintenance by providing a nutritive and immunoprivileged environment (1). Aberrant decidualization hampers embryo implantation, resulting in infertility and adverse pregnancy outcomes, including recurrent implantation failure, recurrent pregnancy loss and pre-eclampsia (2–4). Decidual transformation is associated with increased cellular size, rounded nucleus and enhanced nucleolar complexity, and augmented lipid droplet accumulation (1). DSCs secrete prolactin (PRL) and insulin-like growth factor binding protein-1 (IGFBP-1), which are markers of decidualization. Decidualization represents a multifaceted process regulated by a myriad of factors, particularly the intricate interplay of estrogen and progesterone signaling pathways. These pathways, along with their downstream molecules and associated transcription factors, drive dramatic changes in gene expression (5).

Concurrently, during decidualization, the differentiation of stromal cells is accompanied by significant metabolic changes, with glucose serving as a primary energy source (1). Glucose metabolism primarily comprises two key steps: glycolysis and the tricarboxylic acid cycle (TCA cycle). In most cellular physiological processes, the TCA cycle is the predominant source of ATP. However, during decidualization, endometrial stromal cells exhibit characteristics of the Warburg effect (6). The Warburg effect refers to the phenomenon where cancer cells, even under non-hypoxic conditions, preferentially produce lactate from glucose via glycolysis, rather than through oxidative phosphorylation (7). Glycolysis, rather than the TCA cycle emerges as the primary energy source fueling decidualization, with key enzymes within the glycolytic pathway assuming pivotal roles in orchestrating this biological process. Accelerated glucose flux and increased glucose consumption are essential for ESC differentiation to cope with increased energy demands effectively (8).

Aberrant glucose metabolism, characterized by dysregulated glucose uptake or glycolysis in human endometrial stromal cells (hESCs), contributes to decidualization deficiencies and subsequent embryo implantation failure (9, 10). Beyond the conventional focus on glucose transporters and enzymes associated with carbohydrate metabolism, recent research has unveiled a burgeoning body of evidence underscoring the significance of glucose metabolism-related substances in driving metabolic reprogramming and, consequently, influencing the decidualization process (11, 12). In this review, we endeavor to elucidate the intricacies of glucose catabolism regulation during decidualization, with a particular emphasis on the role of metabolic byproducts. By summarizing recent advancements in the field, we aim to provide an overview of the interplay between glucose metabolism and decidualization (**Figure 1**).

## Glucose uptake and decidualization

Sufficient glucose availability is paramount for the decidualization process, while a diminished glucose environment impairs this critical transformation. Glucose uptake, the first step in glucose utilization, is mediated by a family of glucose transporters (GLUTs), the solute carrier 2A (SLC2A) family. All SLC2As have 12 membrane-spanning helices and several conserved sequence motifs, yet they exhibit tissue-specific distribution, kinetics, and substrate specificity (13). Among these, GLUT1, GLUT3, GLUT4, and GLUT8 are the prominent subtypes of glucose transporters found in the human endometrium. In hESCs, mRNA expression of GLUT1 undergoes dynamic changes during menstrual cycle, showing a significant increase in the mild secretory phase, which is further elevated during decidualization (14). MiR-140-5p downregulates GLUT1 mRNA and protein expression in hESCs leading to reduced glucose uptake and impaired decidualization by activating cell apoptosis. This can potentially impede embryo implantation, and subsequent placental development, both of which are associated with preeclampsia (15). Studies have demonstrated that progesterone facilitates GLUT1 expression by binding to progesterone receptor (PGR) and targeting downstream insulin receptor substrate 2 (IRS2), leading to downstream activation of mitogen-activated protein kinase (MAPK) and phosphatidylinositol-3 kinase (PI3K/AKT) pathways, which critically support the human endometrial decidualization process to facilitate pregnancy (16–18). Furthermore, epigenetic modifications such as histone-H3 lysine-27 acetylation (H3K27ac) have been implicated in the upregulation of GLUT1 mRNA expression, thereby enhancing glucose uptake during decidualization (19, 20). Notably, extracellular vesicles (EVs) carrying GLUT1 cargo proteins secreted by hESCs was identified as promoting glucose uptake, supporting and advancing the decidualization process (21) (**Figure 1**).

In contrast to the variable expression pattern of GLUT1, GLUT3 mRNA expression maintains a constant level in hESCs throughout the menstrual cycle and decidualization (13, 22). GLUT3 knockout mice arrest early embryonic development due to apoptosis of ectodermal cells (23). Nie et al. found that downregulated of GLUT3 protein level in endometrial epithelium by progesterone-induced miR-152 alters the glucose concentration, thus led to impaired embryonic development and implantation (24). Further investigation into the function of GLUT3 in endometrial stromal cells is needed. GLUT4, an insulin dependent glucose transporter, shows a slight decrease in the secretory endometrium compared to proliferative endometrium (25). Dysregulation of GLUT4 disrupts glucose uptake via mediating insulin resistance of endometrial cells, which may hamper the process of decidualization in women with hyperinsulinemia, insulin resistance, or polycystic ovary syndrome (PCOS) (18, 26–28). Intracellularly expressed GLUT8 acts as a sensor for various metabolites, playing a crucial role in cellular metabolic homeostasis (29–31). During decidualization GLUT8 mRNA levels increases without any change in protein abundance (14). Notably, GLUT8 knockout female mice exhibit incomplete decidualization (32). Another glucose transporter of interest is sodium-glucose transporter 1(SGLT1), a high-affinity Na+-coupled glucose transporter. SGLT1 is implicated in facilitating decidualization through glucose uptake, and its abnormal expression may contribute to recurrent pregnancy loss (33). Overall, glucose transporters exert a pivotal role in glucose uptake and promoting decidualization, thus facilitating the establishment of pregnancy. Investigations of dysregulated mechanism of glucose transporters provide novel clinical therapeutic targets.

## Glycolysis and decidualization

Once incorporated into the cells, glucose is subsequently degraded by several pathways: lactate production, or oxidation by the Krebs cycle and the respiratory chain in the mitochondria to provide energy as ATP, or glycolytic bypass metabolic pathways. Glucose metabolism undergoes drastic changes during decidualization. DSCs, to some extent, exhibit Warburg-like metabolic characteristics, such as enhanced extracellular acidification rate (ECAR) indicative of increased glycolysis (8), to meet rapidly growing energy demands. Moreover, blockage of any enzyme of glycolysis can attenuate decidualization process. Metabolic byproducts related to glucose metabolism, such as pyruvic acid, acetyl-CoA, and lactate, has also been proved to participate in the regulation of decidualization. Overall, here we precisely describe the metabolic reprogramming observed during decidualization and underscore the intricate interplay between metabolic pathways and cellular processes.

### HK2 and decidualization

Hexokinase (HK) is the first rate-limiting enzyme of glycolysis catalyzing the conversion of phosphorylate glucose to glucose-6-phosphate (G6P). Mammals possess five HK isozymes, namely HK1, HK2, HK3, glucokinase (GCK), and hexokinase domain-containing 1 (HKDC1), with HK2 being the most extensively studied in glycolysis (34). HK2 stimulates glucose uptake and lactate production in hESCs (35). Downregulated mRNA and protein levels of HK2 via miR-6887-3p suppresses glycolysis, impairing decidualization of ESCs (35) (**Figure 1**).

### PFK1 and decidualization

Phosphofructokinase-1 (PFK1) catalyzes the second committed step of glycolysis, converting fructose-6-phosphate (F6P) into fructose-1,6-bisphosphate (FBP). This enzyme exists in three isoforms: PFK-M (muscle-specific), PFK-P (plasma-specific), and PFK-L (liver-specific), each exhibiting tissue-specific localization in accordance with distinct energy metabolism requirements (36). PFK1 is allosterically activated by 6-phosphofructo-2-kinase/fructose-2, 6-bisphosphatase 3(PFKFB3), which converts fructose-6-phosphate (F6P) to fructose-2,6-bisphosphate (F2,6P). A recent study showed that steroid receptor coactivator-2 (SRC-2) accelerates the glycolytic flux by inducing PFKFB3 mRNA expression to provide the necessary bioenergy and biomass to meet the demands of a high proliferation rate observed in hESCs prior to their differentiation into decidual cells (8). In humans, DSCs exhibit elevated levels of PFK1 and reduced levels of fructose bisphosphatase 1 compared to ESCs, resulting in the accumulation of FBP within DSCs. FBP promotes decidualization, trophoblast invasion, and maternal-fetal tolerance by inducing decidual COX-2+ macrophage differentiation (37). Additionally, FBP fosters a feedback loop involving phosphofructokinase-1 (PFK1), PI3K/Akt, and PFK2/PFKFB3, thereby promoting aerobic glycolysis and sustaining the Warburg effect in cancer cells (38), and further investigation is needed to determine whether this effect exists during decidualization (**Figure 1**).

### PKM2 and decidualization

Pyruvate kinases (PKs) are the second rate-limiting enzymes involved in glycolysis. Four isoforms of PKs have been identified: liver-type PK (PKL), red blood cell PK (PKR), and muscle isozymes M1 and M2 (PKM1 and PKM2, respectively) (39). Among these, PKM2 emerges as a pivotal player in glycolysis in the endometrium and the maintenance of the decidualization process. Upon the initiation of decidualization, PKM2 mRNA and protein expression experiences a significant upregulation *in vivo* and *in vitro*, and studies in mice have shown that PKM2 knockdown or inhibition in the uterus results in aberrant decidualization (40). Consistently, another study demonstrated that abnormal PKM2/PKM1 alternative splicing results in enhanced PKM1 and diminished PKM2 mRNA expression, attenuates decidualization, and may contributed to repeated implantation failure (41) (**Figure 1**).

### Metabolites, epigenetic modification and decidualization

Metabolites of glucose metabolism play a crucial role in the regulation of decidualization process. The levels of lactic and pyruvic acid in hESC exhibit variability across different studies. One *in vitro* study reported no differences in the levels of lactic and pyruvic acid, but the mRNA expression of pyruvate dehydrogenase enzyme 1 alpha (PDHA1) and lactate dehydrogenase A (LDHA) is upregulated in decidual cells compared to non-decidual cells (22). The possible explanation for this finding is the rapid redirection of pyruvic acid towards acetyl-CoA and lactic acid production following pyruvate synthesis. However, several studies have demonstrated that lactic acid level produced by Warburg-like glycolysis is elevated after decidualization (6, 15, 41). Lactate level at the implantation sites was increased in mouse decidua. Lactate is able to promote the proliferation of the undifferentiated cells and differentiation ability of decidualizing cells (6). During *in vitro* decidualization of hESCs, mRNA expression of GLUT1, LDHA and monocarboxylate transporter 4 (MCT4) elevate at the same time. GLUT1 knockdown markedly reduced glucose uptake and lactate production, and reduced the levels of decidualization markers (15). Lactic acid may act as signal to alter cell functions of the endometrium, thereby enhancing endometrial receptivity and the initiation of implantation (42) (**Figure 1**).

In cancer, lactate, an energy source and metabolic by-product, can shuttle between glycolytic tumor cells and oxidative tumor cells, thereby promoting tumor occurrence and development (43). Similarly, glycolytic cells and oxidative cells may establish intracellular connections and synergistic metabolism by lactate shuttle in the endometrium during decidualization. Furthermore, Zhang et al. described for the first time that lactate act as a substrate for lactylation, which can modulate histone lactylation at the H3K18 site and directly regulate gene expression (44). Subsequent investigations have revealed numerous lactylation events occurring on non-histone proteins such as transcription factors (45), or writer protein of m6A METTL3 (46). Particularly, enzymes involved in metabolic pathways such as the tricarboxylic acid cycle, carbohydrate, amino acid, fatty acid, and nucleotide metabolism, can also be lactylated (47). Lactate produced by glycolysis increases the lactylation level of PKM2 protein, which inhibits its tetramer-to-dimer transition, reducing nuclear distribution and enhancing its pyruvate kinase activity in the cytoplasm (48). Lactylation can impact the activity of glucose metabolism-related enzymes, suggesting that lactylation may act as a feedback regulator of glucose metabolism. *In vivo*, lactylation at H4K12la promotes expression of Hif1α and enhances endometrial glycolysis, and in turn forms an H4K12la-Hif1α-glycolysis feedback loop to drive decidualization (12). Enhanced lactate production resulting from upregulated glycolysis further induces endometrial H3K18 lactylation and regulates redox homeostasis and apoptotic balance, thereby facilitating endometrial remodeling to ensure successful implantation (49). However, the specific impact of lactylation on non-histone proteins in the decidualization process remains unclear.

Notably, pyruvate dehydrogenase kinase 4 (PDK4) mRNA expression exhibits significant upregulation during *in vitro* decidualization. PDK4 functions by attenuating the activity of pyruvate dehydrogenase complex (PDC) through PDHA1, thereby promoting the preference for pyruvate dehydrogenation and accelerating the Warburg effect (50, 51). The inactivation of PDHA1 leads to down-regulation of IGFBP1 and PRL, indicating compromised decidualization processes. Furthermore, PDHA1 has been observed to translocate to the nucleus, leading to increased acetyl-CoA and the metabolite pool within the nucleus. This translocation enhances histone H3 acetylation, facilitating chromatin remodeling and genome activation (52, 53). In hESCs, genome-wide analysis found that histone modifications regulate gene expression by altering chromatin structure to facilitate decidualization (54). CCAAT/enhancer binding protein beta (C/EBPbeta) works with cofactors, such as p300, which is constitutively expressed in human endometrium. CBP/p300 binds to the promoter and enhancer regions of target DNA sequences to induce H3K27ac and opens the chromatin structure, which involved in the regulation of IGFBP1 and PRL during decidualization (55–57) (**Figure 1**).

### TCA cycle

Pyruvate can be metabolized to either lactate or acetyl-CoA. Then, acetyl-CoA enters the TCA cycle. Alternatively, lactate can be converted back to pyruvate for conversion to acetyl-CoA and then go into the TCA cycle. Interestingly, the overall TCA pathway seems to be less active during decidualization (22). Moreover, glutamine metabolism assumes a critical role beyond serving as an anaplerotic influx into the TCA cycle for carbon supply. The production of α-ketoglutarate supports successful decidualization through epigenetic regulation of histone modifications and energy provision (58). Succinate dehydrogenase complex iron sulfur subunit (SDHB) is a critical subunit of succinate dehydrogenase, which is part of complex II in the mitochondrial electron transport chain and is responsible for the conversion of succinate to fumarate. Decreased DNA methylation of SDHB elevates SDHB mRNA expression in the chorionic villi, which reduces overall succinate accumulation and risk of recurrent spontaneous abortion (59). Therefore, metabolites involved in the TCA cycle, such as citrate, and α-ketoglutarate, have been shown to play crucial roles in decidualization and pregnancy maintenance.

### Glycolytic bypass metabolic pathways during decidualization

#### Pentose phosphate pathway

PPP pathway is indispensable for glucose metabolism in decidualization. Blockade of glucose-6-phosphate dehydrogenase (G6PDH), the rate-limiting step in PPP, impairs decidualization, as does glucosamine (competitive inhibitor of G6PDH) (60). This finding has been reinforced by using pharmacologic inhibitors of PPP, 6-aminonicotinamide or dehydroepiandrosterone (DHEA) in both vitro and *in vivo* studies (61). Additionally, studies also demonstrate that exogenous nucleoside can rescue decidualization in endometrial stromal cells that have been impaired by ribose-5-phosphate depletion (61). DHEA, is reported to be elevated in a significant proportion of women with PCOS. However, an *in vitro* study reported that supplementation with DHEA increases the mRNA and protein expression of the decidualization markers IGFBP1 and PRL, and may augment endometrial function and improve pregnancy rates (62). Taken together, the mechanism and impact of DHEA on endometrial function and decidualization remain unclear, more investigation is needed.

#### Hexosamine biosynthesis pathway

The hexosamine biosynthesis pathway (HBP) is an important glucose metabolism pathway that synthesizes uridine diphosphate-N-acetyl glucosamine (UDP-GlcNAc), which is subsequently utilized for post-translational modification (O-GlcNAcylation) of intracellular proteins that regulate nutrient sensing and stress responses. Activation of endometrial HBP during the window of implantation exerts profound effects on endometrial cell function and embryo implantation. Zhang’s study elucidates that elevated O-GlcNAcylation impacts endometrial cell function by redirecting glucose metabolic flow, shunting it toward the PPP and HBP. This metabolic reprogramming in ESCs during decidualization satisfies the demands for nucleotides and reducing equivalents while also facilitating metabolic-epigenetic regulation of gene expression, such as aquaporin 3 (AQP3) (63). In addition, glucose metabolism through HBP modulates cytoskeleton changes, thus may influence endometrial receptivity to an implanting embryo via O-GlcNAcylation of Myosin phosphatase target subunit 1 (MYPT1) (64) (**Figure 1**).

#### Serine synthesis pathway

The serine synthesis pathway (SSP), although infrequently studied in endometrial decidualization, has been extensively investigated in cancer cells. 3-phosphoglycerate generated from glycolysis in cancer cells is oxidized by PHGDH (phosphoglycerate dehydrogenase) to 3-phosphohydroxypyruvate, a precursor for *de novo* serine synthesis (65). SSP activation lead to elevated glutathione (GSH) production, cell cycle progression and nucleic acid synthesis, which are essential for cell survival and proliferation especially under nutrient-deprived conditions (66).The relationship between glycolysis and SSP has been described in astrocytes, where glycolytic flux modulates D-serine production through the interaction between serine racemase and a glycolytic enzyme, glyceraldehyde 3-phosphate dehydrogenase (GAPDH) (**Figure 1**) (67).

## Conclusion

In this review, we delve into the functions and regulation of various glucose transporters and key enzymes catalyzing pivotal steps in glucose metabolism, as well as the impact of metabolites on decidualization (**Figure 1**). While mounting evidence suggests that augmented glucose utilization plays a central role in endometrial decidualization. However, the precise regulatory mechanisms by which glucose metabolism and related enzymes affect decidualization in endometrial stromal cells remain elusive. From a clinical perspective, elucidating these regulatory pathways may offer molecular and cellular insights into the physiological and pathological mechanisms of pregnancy-related disorders associated with impaired metabolic homeostasis. Furthermore, identifying novel metabolic targets could pave the way for the development of innovative clinical approaches aimed at diagnosing and treating these reproductive disorders effectively.

## Glossary

cAMP: cyclic adenosine monophosphate
DSCs: decidual stromal cells
ECAR: extracellular acidification rate
ESCs: endometrial stromal cells
F6P: frucotose-6-phosphate
FBP: fructose-1,6-bisphosphate
G6P: glucose-6-phosphate
GLUTs: glucose transporters
H3K27ac: histone-H3 lysine-27 acetylation
hESCs: human endometrial stromal cells
HK: Hexokinase
IGFBP-1: insulin-like growth factor binding protein-1
IRS2: insulin receptor substrate 2
LDHA: lactate dehydrogenase A
MAPK: mitogen-activated protein kinase
MCT4: monocarboxylate Transporter 4
PCOS: polycystic ovary syndrome
PDHA1: pyruvate dehydrogenase enzyme 1
PDK4: pyruvate dehydrogenase kinase 4
PFK1: Phosphofructokinase-1
PFKFB3: 6-phosphofructo-2-kinase/fructose-2, 6-bisphosphatase 3
PGR: progesterone receptor
PI3K/AKT: phosphatidylinositol 3 kinase/protein kinase B
PKs: pyruvate kinases
PRL: prolactin
SDHB: succinate dehydrogenase complex iron sulfur subunit
SGLT1: sodium-glucose transporter 1
TCA cycle: tricarboxylic acid cycle
